# The challenges of widening access to the medical profession: how to facilitate medical careers for those at a genuine disadvantage

**DOI:** 10.15694/mep.2018.0000277.1

**Published:** 2018-12-07

**Authors:** Nana Sartania, Graham Haddock, Mark Underwood

**Affiliations:** 1 University of Glasgow

**Keywords:** Widening Participation, Outreach, Selection, Admissions

## Abstract

This article was migrated. The article was marked as recommended.

Widening Participation (WP) for medical school entry has been politically encouraged to ensure access and participation for underrepresented groups rarely able to gain access to this high demand profession. Those who reside in the 20-40% most deprived postcodes in Scotland (SIMD20) are much less likely to apply for medical school entrance, and even less likely to succeed. The National Reach programme in Scotland aims to rectify the existing situation by encouraging and supporting students from working class backgrounds to apply to high demand courses, including medicine, and has achieved great success in helping pupils from target secondary schools to gain a place in Glasgow Medical School. However, some of the Reach students have similar demographics to the rest of the medical school class and arguably do not genuinely belong in the target group. To address this, a second flag based on SIMD20 residence was employed. However, applying more than one WP flag - while substantially improving the targeting of this programme and helping those who truly are multiply deprived - reduces the Reach-eligible applicant pool to the point of undermining the high WP targets imposed on Universities. But using only a single criterion of MD20 residence or school progression rate would unfairly advantage some pupils that are actually not disadvantaged. Ideally, individualised indicators such as eligibility for Free School Meals, possession of an Educational Maintenance Allowance or receipt of a UKCAT bursary, would complement residential data and school progression rates. This paper reflects on the evolution of the admissions practices in our medical school designed to comply with the targets, but also create a medical workforce reflecting the population it serves.

## Introduction: recognition of the need for wider participation

Widening participation (WP) is a hot topic on the political agenda in a number of countries - in the UK this is to a large extent about underrepresentation of white males from working class background in the top professions ( Lambert, 2018; Sullivan, 2010; Baars, 2015; Adams, 2018); in Canada it is about participation of the indigenous population in Higher Education (HE) ( Collier, 2010), while in Germany it is reported that children of academics are more likely to apply and succeed in getting a place on the medical course ( Simenroth-Nayda, 2015). Family income, social or cultural capital (belonging to a network that enables social mobility) ( Nicholson, 2015; Wilkes, 2018), the level of parents’ education ( Simenroth-Nayda, 2015; Powis, 2007), and the geographical location ( Cooper, 2017) all influence a young person’s opportunity, and decision, to pursue medicine as a career. Yet it is now universally recognised that it is important that the medical workforce demographic resembles the population it serves in order to provide quality care through shared experiences free of bias, improving the doctor - patient relationship, including trust and understanding Torres, 2018; Berger, 2008). The debate is about how we can bring this about.

## Addressing the problem: the Reach and SWAP programmes

According to Universities Scotland, school leavers from the 20-40% most deprived postcodes in Scotland (SIMD20/40, Scottish Index of Multiple Deprivation, a measure of socio-economic disadvantage) are 5-10 times less likely to qualify for university entrance (US, 2012), let alone for the undergraduate (UG) medical course with its more demanding academic admissions requirements. Recognition of the need to increase access for, and participation of, underrepresented groups in Scottish Universities led to the establishment of the National Reach outreach programme in 2010. This programme aims to raise the aspirations of underrepresented groups while they are still at school and provide them with practical help on accessing specific high-demand professions: Medicine, Veterinary Medicine, Law and Dentistry. The initiative is shared by five universities in Scotland (Glasgow, Edinburgh, St-Andrews, Dundee and Aberdeen), and is funded by the Scottish Funding Council (SFC) with the aim of addressing the existing disparity in terms of representation of students from working class backgrounds on these courses, mainly due to the attainment gap among the pupils.

From the outset of the programme the UG Medical School in Glasgow has engaged with an increasing number of secondary schools in the West of Scotland whose progression rate to HE is below the national average (of currently 35%). 95 such schools send pupils from years 4 to 6 of secondary education (S4-S6) to take part in the programme, which offers in-school sessions on essay writing, study skills and critical appraisal of a given medical topic, as well as a week-long Summer School. The Summer School aims to offer impartial, easily accessible information regarding university learning and teaching, offers an opportunity to experience lectures and small group teaching sessions. It also provides tailored support with the application process, which includes preparing the pupils to sit the UK Clinical Aptitude Test (UKCAT) and the interview. Moreover, during the week pupils have access to simulated clinical experience and discuss various career pathways that are possible with a medical degree.

It was hypothesised that working in clusters will allow pupils to meet others from similar backgrounds and be part of a network of like-minded people they can rely on when/if they make the transition from school to university. This helps the Reach participants to overcome barriers on the challenging road to successful applications, and, after that, aids student retention. Although the overarching ethos of the programme is similar, each University engages with schools with different degrees of intensity.

After the successful pilot, in 2012, the Glasgow Medical School UG selection panel decided to use contextual admissions for those who completed the Reach programme. Student demographics, verified centrally on receipt of applications were taken into consideration when downward flexibility on UKCAT (10% uplift in scores) or academic entry requirements (two grades lower accepted, should these be below the published standard entry requirements) were applied. As such, we were allocating 16-18% of the total available places to students coming from target schools, as a result of a robust implementation of the Reach programme.

In addition, the UG medical school in Glasgow has been working with further education colleges to run the Scottish Wider Access Programme (SWAP), supporting access to university for adult learners. This programme is aimed at mature applicants and attracts a wide range of those who did not achieve the grades, and/or have the opportunity, to enter the medical profession on completing their secondary education. After completing a one-year access programme and passing it ‘with merit’, they must sit the UKCAT and submit themselves for interview. Another category of more mature applicants that UG Medical School actively tries to encourage are those with care experience (care givers/care experienced) and asylum seekers.

## Fine-tuning of targeting to the right student cohort

There is a direct correlation between the progression rate to HE for a secondary school and the proportion of pupils in that school residing in MD20/40 postcodes ( McKenna, 2016; Croll, 2016). Indeed, a school may have a low progression rate exactly because of low attainment of its MD20/40 pupils, who will not progress to HE in high numbers. This would logically suggest that it is the pupil’s residential postcode, and expectations from home, rather than the school attended, that provides the disadvantage. This rationale is echoed in the Scottish Government’s target that recommends that at least 20% of full-time first-degree entrants to Scottish universities should come from the 20% most deprived postcode areas ( Somerville, 2017). Although it can be argued that targeting by lower progression school is valid as these schools are predominantly populated by pupils living in MD20/40 areas, the student’s demographic must still be carefully weighed before using contextual data for their admissions because not all students in such schools are from genuinely deprived backgrounds.

For example, depending on the criteria used (parental experience of HE, postcode residence or Socio-Economic code, an occupationally based classification reflecting parental socio-economic background), 60-80% of the Reach students admitted between 2012-2015 had demographics that were similar to the rest of the (non-Reach) class and could thus be argued not to genuinely belong in the target group at all. Therefore, applying more than one widening participation flag will substantially improve the targeting of this programme and help those who truly are multiply deprived. Using a blanket measure of MD residence or school targeting will give an unfair advantage to those who attended the low progression schools but didn’t reside in MD20/40 postcodes, while it is also likely that we would fail to admit those who by postcode residence would have been in our target group and should have benefitted from an adjusted offer.

## Recent University of Glasgow initiatives related to widening access

In order to address this, the medical school has extended the same policy of contextual admissions to those who matriculated between 2012 and 2016 and did reside in a target postcode area but did not take part in Reach outreach programme. By amending the policy, we have made the process transparent and much fairer for all MD20/40 residents. Ideally, all applicants from the target postcodes should have been through Reach programme as well and benefited from this intervention. However, in order to meet the Scottish government’s 2030 target of 20% HE entrants from SIMD20 postcodes, the University of Glasgow may need to extend the current targeting matrix to SIMD20 residents in high progression schools as well, as pupils living in MD20 areas are statistically less likely to progress to HE, as they live in an area of high deprivation and have a low expectation to go to University. At the same time, it must be recognised that attending low progression schools will put a pupil at a disadvantage regardless of residence postcode because it increases the likelihood of them not achieving the grades necessary to enter the highest-demand courses in HE. Thus, although there is justification for applying the WP flag for each condition, we must conclude that by applying just one criterion it is certain that a significant percentage of ‘false positives’, i.e. applicants that are not truly multiple-deprived will be given tariff discounts that their peers are not entitled to.

The other option to increase the pool of genuinely disadvantaged applicants would be to engage with low HE progression secondary schools earlier on, in order to raise the aspirations of SIMD20 pupils. There are encouraging examples of engagement from our students working with pupils in nursery schools and primary schools, who manage to boost aspiration among the pupils, although it is recognised that a sustained effort is required to not only kindle interest in the medical profession but to nurture the enthusiasm and interest for it to last until the application stage.

For the same reason, the Glasgow medical school targets S3 pupils from MD20 areas across the West of Scotland, in order to engage in a meaningful conversation with them before they select their S4 subjects; this ensures that pupils have access to the right information at the right time and choose the appropriate subjects that enables them to pursue a medical degree. The event is advertised via schools within 12 Local Authorities in the West of Scotland and provides a platform for information delivery from senior academics, clinicians, admissions officers and welfare staff. The well-attended event is supported by the Glasgow University Widening Access to Medicine Student Society (GUWAMS) as well as graduates from WP background, currently junior doctors. Most importantly, however, these events are designed to target not just pupils but their parents as well. It is widely accepted that an attitudinal change is required among the parents of pupils from lower socio-economic backgrounds if we want to attract the right group. We hypothesised that events that target both pupils and their parents, the main influencers of the decision-making process whether to go to HE, would significantly improve our chances of engaging the target audience.

In Glasgow, the S3 event paves the way for pupils to enrol into the Reach outreach programme that eventually prepares them for entry to the profession. The medical school recently graduated the first cohort of Reach medics who themselves are tremendous role models for the next generation of WP applicants. The University and the medical school provide full support to those who want to succeed - first by raising aspiration, then supporting them through the application process, applying agreed tariff flexibility, and finally providing mentoring to ensure they succeed on the course. It is our belief that in this way we will see an increase in genuinely disadvantaged applicants to the medical school, with improved grades and motivation.

More recently in 2017, the Scottish Government funded a pilot project (the Glasgow Access Programme, GAP) aimed at home students from MD20 postcodes, designed to provide the adequate knowledge in key subjects that is necessary at entry level. 20 students were recruited to a one-year pre-medicine gateway course for which the entry requirements were lowered significantly (AABB at Scottish Higher vs standard AAAAA or AAAABB) and those who qualified were offered a place after interview. The condition of acceptance was for the students to perform well in the GAP course, at the end of which they transition into year one of UG medicine without further interview or the need to sit the UKCAT. The success of the first cohort (95% entered the medical curriculum after successful completion of GAP) demonstrates that the approach is right and can generate the change needed in order to further widen participation of the able pool of applicants from non-traditional backgrounds.

## Outlook

Ideally, a combination of externally validated individualised measures such as Free School Meal (FSM ^1^), Educational Maintenance Allowance (EMA ^2^) or receipt of UKCAT bursary ^3^ reflecting an individual’s circumstances, would complement SIMD20/40 data and school progression rates that change over the time. The Sutton Trust report *Admissions in Context* strongly recommends a greater use of individual or household measures ( Crawford, 2017) that could allow more effective targeting and intervention to those applicants experiencing disadvantage and enable bespoke contextualised admission decisions to be made for offers of entry by the medical schools. We should also work towards a harmonisation of access-related programmes/criteria for all Scottish Medical Schools. While it will take time for these programmes to translate in a diverse medical workforce representing all of society, we believe that current strategies have finally set us on a course to achieving that aim.

## Footnotes

**^1^FSM** is a statutory benefit available to school-aged children from families who receive other qualifying benefits and who have been through the relevant registration process

**^2^EMA** is a financial scheme applicable to students and those undertaking unpaid work-based learning in the United Kingdom (except England) and aged between sixteen and nineteen whose parents had a certain level of taxable income.

**^3^UKCAT** bursary - Applicants who have independently verified financial disadvantage, either as an individual or at a household level receive a bursary to sit this test prior applying to medical schools.

**UKCAT** - UK Clinical Aptitude Test

## Take Home Messages

• The Reach programme to widen participation of non-traditional applicants in Glasgow has targeted pupils from low progression schools and has increased successful WP applications.
• Adding a second condition, MD20 residence, while improving targeting of these programmes, decreases the applicant pool for WP schemes.
• Extending the targeting matrix to all applicants from deprived postcodes made the process transparent and fairer for all MD20/40 residents and helped meet WP target.
• Engaging with pupils from early secondary school years to nurture aspiration paves the way for pupils to enrol into the Reach outreach programme.
• Gateway programmes (like the Glasgow Access Programme) attract able and successful applicants from non-traditional backgrounds, residing in MD20 areas.

## Notes On Contributors

Nana Sartania, PhD, M.Ed is a Deputy Director of Admissions and Senior Lecturer in the Undergraduate Medical School in University of Glasgow. Her research interests are concerned with the predictive validity of the admissions criteria currently used in the UK and how the use of contextual data in the admissions process impacts on the school’s efforts to widen access to the medical education locally.

Graham Haddock, MD, MBChB, is Honorary Clinical Associate Professor and Admissions Lead in undergraduate Medical School of the University of Glasgow. His focus is on widening participation of underrepresented groups. Mr Haddock is a paediatric surgeon at the Royal Hospital for Sick Children in Glasgow.

Mark Underwood, MD, MBChB, is a Consultant Urological Surgeon at the Glasgow Royal Infirmary and Deputy Director of Admissions for Glasgow Medical School.

NS was involved in the original design, data collection and writing of the first draft of the paper. GH and MU contributed to the discussions and reviewed the paper. All authors read and approved the final manuscript.
