# Supplementary material for: The challenges of widening access to the medical profession: how to facilitate medical careers for those at a genuine disadvantage
Source: MedEdPublish (2016). 2019 Jul 5;7:277. Originally published 2018 Dec 7. [Version 2] doi: 10.15694/mep.2018.0000277.2 (PMC10711983; doi:10.15694/mep.2018.0000277.2)
Supplement: Peer review history

# Reviewer report: Reviewer response for version 2

**DOI:** 10.21956/mep.20237.r31369

**Published:** 2020-01-01

**Authors:** P Ravi Shankar

**Affiliations:** 1 American International Medical University

This review has been migrated. The reviewer awarded 4 stars out of 5 Over the last year (2019) there have been many papers in the journal discussing the issue of widening access to medical schools. This is a well-written paper which discusses the issue of widening access to the undergraduate medical program at the University of Glasgow. I am not sure if the successful applicants also receive financial and other support during their course of study. Also are there any service obligations for graduates of the program? Many of the lessons outlined in this paper could be applied to other countries and locations though, there will also be significant differences.

Reviewer Expertise:

NA

No decision status is available

# Reviewer report: Reviewer response for version 2

**DOI:** 10.21956/mep.20237.r31366

**Published:** 2019-07-15

**Authors:** Jennifer Cleland

**Affiliations:** 1 University of Aberdeen

This review has been migrated. The reviewer awarded 2 stars out of 5 An interesting paper reporting on a local approach to widening access. The paper would have benefited from more grounding in the wider literature on the limits of the markers used for WA admissions (such as SIMD) rather than just quoting the Sutton Trust report. Doing so may have also made this more accessible to people in other contexts/countries. There is also some recent data on these markers from Paul Lambe which would have been relevant to this paper - as it casts doubt on the utility of the so-called "basket of contextualised data" that the authors are proposing.

Reviewer Expertise:

NA

No decision status is available

# Reviewer report: Reviewer response for version 2

**DOI:** 10.21956/mep.20237.r31368

**Published:** 2019-07-11

**Authors:** Ken Masters

**Affiliations:** 1 Sultan Qaboos University

This review has been migrated. The reviewer awarded 4 stars out of 5 This is a revised version of the authors’ previous submission. The authors have adequately addressed my concerns.

Reviewer Expertise:

NA

No decision status is available

# Reviewer report: Reviewer response for version 2

**DOI:** 10.21956/mep.20237.r31367

**Published:** 2019-07-11

**Authors:** Louise Alldridge

**Affiliations:** 1 University of Plymouth

This review has been migrated. The reviewer awarded 5 stars out of 5 This is my second review. This paper outlines the commonly encountered problems of widening participation in medicine but is also more widely applicable to accessing Elite Universities as well as other over-subscribed and professional programmes such as Veterinary Science, Law etc.It openly addresses one of the biggest issues in ensuring WP interventions are reaching truly disadvantaged students. The paper has developed the deeper implications and related these to the three forms of capital and the reproduction of society.

Reviewer Expertise:

NA

No decision status is available

# Reviewer report: Reviewer response for version 1

**DOI:** 10.21956/mep.18989.r27220

**Published:** 2019-03-23

**Authors:** Paul Tiffin

**Affiliations:** 1 University of York

This review has been migrated. The reviewer awarded 5 stars out of 5 This is a timely and thoughtful article. The authors rightly raise some of the practical challenges of identifying and defining WP candidates, correctly stressing the role of individual-level markers. They also provide a good example of an initiative addressing some of the very real disadvantages faced by under represented groups. My only suggestion is that most articles on this topic should first stress that the whole WP issue in the UK is an epiphenomena of the increasingly marked societal inequalities, especially in respect to income and education. Therefore universities are under pressure to fix a problem that has been caused by governmental policies that have exacerbated inequalities for some period now.

Reviewer Expertise:

NA

No decision status is available

# Reviewer report: Reviewer response for version 1

**DOI:** 10.21956/mep.18989.r27219

**Published:** 2019-02-10

**Authors:** patrick west

**Affiliations:** 1 retired

This review has been migrated. The reviewer awarded 4 stars out of 5 This is an interesting and well written position paper on the important issue of widening access to medical school among students from disadvantaged backgrounds. It is descriptive both in respect of the overall problem and in respect of the school-based programmes to increase rates and success of medical school application. The authors clearly identify the basic problem with the current (school- based) targeting, which results in over identification of non-deprived pupils, and make a persuasive case for the inclusion of individual and/or household factors to reduce the number of ‘false positives’. The only criticism I would make is that the authors should acknowledge more clearly that the programme is based in Scotland and might not generalise to other educational systems. Readers from outside Scotland are likely to find references to specific stages of secondary school (S3, S4 etc) difficult to understand without more context.

Reviewer Expertise:

NA

No decision status is available

# Reviewer report: Reviewer response for version 1

**DOI:** 10.21956/mep.18989.r27218

**Published:** 2019-01-10

**Authors:** Enam Haque

**Affiliations:** 1 University of Manchester

This review has been migrated. The reviewer awarded 4 stars out of 5 Really interesting paper, covering the invaluable Glasgow Reach Programme. Adding a second condition is an important step, to ensure authenticity of pupils applying to widening participation programmes. I would love to see a comparative study with an English based medical school programme, to see if what the differences are.Enam HaqueChair, National Medical Schools' Widening Participation Forum

Reviewer Expertise:

NA

No decision status is available

# Reviewer report: Reviewer response for version 1

**DOI:** 10.21956/mep.18989.r27222

**Published:** 2018-12-24

**Authors:** Ken Masters

**Affiliations:** 1 Sultan Qaboos University

This review has been migrated. The reviewer awarded 3 stars out of 5 An interesting read dealing with issue of ensuring a wider intake of students.Some comments:• The focus of the paper is on one country only. This should have been reflected in the title (even though some of the lessons learned may be more internationally applicable). It should similarly be taken into account in the body of the paper, where references to “MD20/40 postcodes” and “S3 pupils” are, frankly, meaningless to readers not familiar with Scotland.• Even though the demographic indicators are somewhat specific to the country, the paper does highlight the danger of using a single demographic indicator to identify a group of people. This is especially dangerous in the case of postal codes, an indicator that can easily be manipulated by candidates desperate to get into medical school. (For example, it would have been interesting to know how many of the students from those areas had moved into those areas shortly before applying to medical school, a practice not unheard of in other countries).• This broader danger, however, needs to be read into the paper by the reader. I think the paper’s value to the general reader could have been increased if the authors had returned to reflect on some of the examples they gave in the opening paragraph dealing with similar issues in other countries.

Reviewer Expertise:

NA

No decision status is available

# Reviewer report: Reviewer response for version 1

**DOI:** 10.21956/mep.18989.r27221

**Published:** 2018-12-16

**Authors:** Louise Alldridge

**Affiliations:** 1 University of Plymouth

This review has been migrated. The reviewer awarded 4 stars out of 5 This paper outlines the commonly encountered challenges of widening participation in medicine.It openly addresses one of the biggest issues and to some extent the ‘elephant in the room’, i.e ensuring WP interventions are reaching and helping truly disadvantaged students. This includes targeting students for interventions and the eventual selection criteria used. This is not an easy mission and is not helped by the criteria with which the government measure success.The paper outlines and reflects on, in an open and honest narrative, the evolution of a strategy to target working class students to ultimately produce a workforce that reflects the population it serves.As such I believe it provides useful information, based on the considerable experience of the authors, for all those working in WP in a medical setting.I only have one recommendation for a small change.In the introduction, the authors collectively defines social and cultural capital as belonging to a network that enables social mobility. These are two different forms of capital that contribute to a person’s habitus (and both very important with regards to accessing a medical career in the medical ‘field’). It would be better to see social and cultural capital defined separately and reference Bourdieu at this point.

Reviewer Expertise:

NA

No decision status is available
